# Oblique Axis Hypothenar Free Flaps: Tips for Harvesting Larger Flaps with Minimal Donor Site Morbidity

**DOI:** 10.1055/a-2008-8671

**Published:** 2023-05-29

**Authors:** Sang Ho Oh, Jae In Chung

**Affiliations:** 1 Department of Plastic and Reconstructive Surgery, Saeson Hospital, Daejeon, Republic of Korea

**Keywords:** fingertip reconstruction, flap design, hypothenar free flap, perforator flaps, soft tissue reconstruction

## Abstract

**Background** Hypothenar free flaps (HTFFs) have been widely used for reconstructing palmar defects. Although previous anatomical and clinical studies of HTFF have been conducted, this technique still has some limitations. In this study, we describe some tips for large flap design that allows for easy harvesting of HTFFs with minimal donor site morbidity.

**Methods** A total of 14 HTFF for hand defect reconstruction were recorded. The oblique flap was designed in the proximal HT area following relaxed skin tension line along the axis between fourth web space and 10 mm ulnar side of pisiform. A flap pedicle includes one or two perforators with ulnar digital artery and HT branch of basilic vein. In addition, innervated HTFF can be harvested with a branch of ulnar digital nerve. Electronic medical records were reviewed to obtain data on patients' information, operative details, and follow-up period. In addition, surgical outcome score was obtained from the patient, up to 10 points, at the last follow-up.

**Results** Mean harvest time was 46 minutes, and two perforators were included in 10 cases. The mean flap area was 10.84 cm ^2^ . There were no problems such as donor site depression, scar contracture, keloids, wound dehiscence, numbness or neuroma pain at donor sites, and hypersensitivity or cold intolerance at flap site, either functionally or aesthetically.

**Conclusion** Palmar defect reconstruction is challenging for hand surgeons. However, large HTFF can be harvested without complications using the oblique axis HTFF technique. We believe our surgical tips increase utility of HTFF for palmar defect reconstruction.

## Introduction

The principle of soft tissue reconstruction in plastic surgery is to replace the defects with the most similar tissues and minimize donor site morbidity. Reconstruction of soft tissue hand defects is technically challenging for hand surgeons. The use of similar and adjacent tissues for reconstruction is important as the characteristics of the dorsum and palmar tissues differ. 1

Historically, the tissue in the hypothenar (HT) area, which exhibits more tissue redundancy than tissues in other areas, has been used for the reconstruction of hand defects. 2 Since 1996, when the HT free flaps (HTFFs) were first introduced following advances in microsurgery and perforator free flaps, it has been used for the effective reconstruction of palmar defects. 3 4 Nowadays, it is used as a sensate flap after confirming the positions of the perforator and the nerve innervating the flap. 1 3 5 6 7 8 We typically select the HTFF for reconstructing the palmar defect because there are more advantages compared with other reconstructive options, it can be harvested with glabrous tissue in one stage under single anesthesia and operative field.

However, some of the disadvantages of the HTFF include a high learning curve due to the small perforator diameter, limited flap size, short pedicles, and scar contractures at donor sites. 9 10 This disadvantage of HTFF makes a limited indication for the reconstruction of palmar defects. Kodaira et al and Yamamoto et al have recently come up with novel flap design and harvest techniques to overcome these shortcomings. 9 11 However, Kodaira and Fukumoto's method is limited by flap harvest size, whereas Omokawa et al's method requires an additional flap for donor site closure. To overcome these drawbacks and allow optimal harvesting of HTFF, we suggest a new concept for large flap design that allows for easy harvesting of HTFF that can be used for the reconstruction of wider defects.

## Patients and Methods

The experimental protocol was approved by the institutional review board of Korea National Institute for Bioethics Policy (P01-202112-21-022). Patients provided informed consent for the publication of the clinical photographs included in this article.

### Patients

In our study, the medical records of a total of 14 HTFF surgeries for hand defect reconstructions were extracted. After the participants were selected, sex, age, operative techniques, flap size, harvest time, injury mechanism, and the number of perforators included in the flap were obtained from the electrical medical records.

### Surgical Techniques

**Fig. 1:**
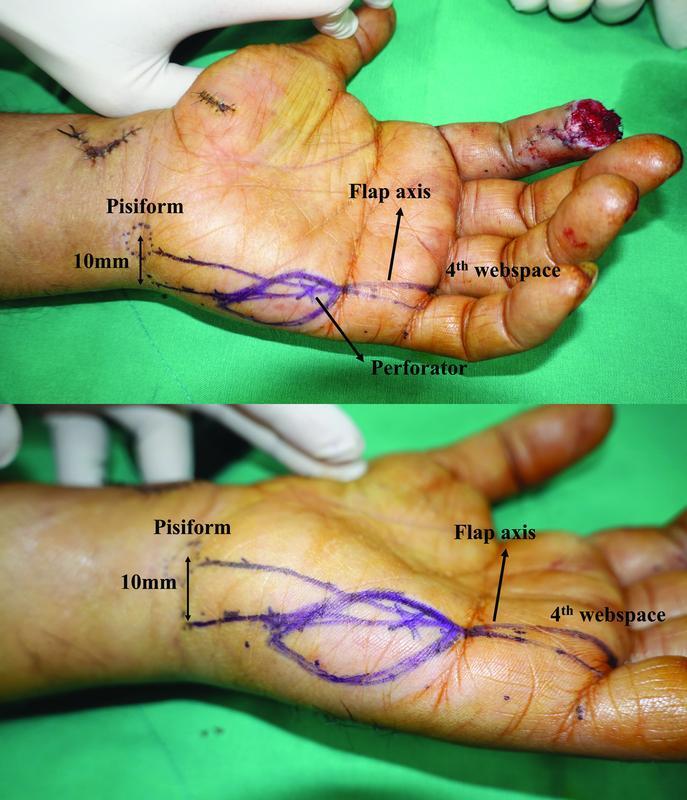
Preoperative flap design. The flap axis indicates the line extending from the fourth web space to a point 10 mm from the ulnar side of the pisiform.

The procedure was performed on patients under brachial plexus block in the supine position by a single plastic surgeon (S.H.O.). First, a line between the midpoint of the metacarpophalangeal (MCP) crease of the little finger and the center of the pisiform was drawn. The dominant perforators are located slightly on the ulnar aspect of this line. One or two dominant perforators were usually found within a distance of 10 mm proximally from the distal palmar crease using a handheld Doppler. The flap was designed along the axis from the fourth web space to a point 10 mm from the ulnar side of the pisiform. The proximal margin of the flap was not passed along the proximal margin of the pisiform bone. The perforator was positioned at one-third of the HTFF ( Fig. 1 ).

**Fig. 2:**
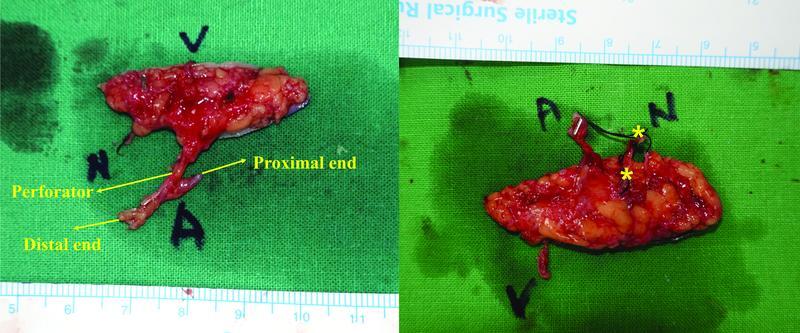
Innervated hypothenar free flap. (Left) One perforator containing both the distal and proximal end of the ulnar digital artery was included in the flap. It can be used as a flow-through type flap. (Right) Two branches of the ulnar digital nerve were included in the flap (asterisk).

After the recipient vessel was prepared, an incision was made on the ulnar side of the flap, and the HT branch of the basilic vein was located in the subdermal layer. To the best of my knowledge, this vein is typically located along the line extending from the ulnar side of the little finger to a point 15 mm from the ulnar aspect of the pisiform. The maximum possible length of this superficial vein was harvested. After an incision was made on the radial side of the flap extending to the deep fat layer, a meticulous dissection was performed to find the perforators. When the perforators were found, the dissection was continued in the retrograde direction to check the site where the perforator branched from the ulnar digital artery (UDA) of the little finger. Since the ulnar digital nerve (UDN) of the little finger usually travels together with and along the UDA, flaps can be harvested by including one or two UDN branches together. Considering the recipient vessel and flap insetting position, the UDA was harvested with 1 to 2 perforators in the distal or proximal direction ( Fig. 2 ).

At the donor site, a Penrose drain was inserted and primary closure was performed. Usually, suprafascial dissection is automatically performed to harvest the HT branch of the basilic vein to facilitate tension-free closure.

### Postoperative Follow-Up

After HTFF, patients are discharged from our clinic at 2 weeks postoperatively if there are no complications. All patients are reviewed in our outpatient clinic at 1, 3, and 6 months postoperatively. Patients lost to follow-up before 6 months were excluded from the present study. At the last follow-up visit, the durability and elasticity of HTFF, flap contour, cold intolerance, and complications at the donor site (scar contractures, donor site depressions, keloid, pain, etc.) were evaluated. In addition, surgical outcomes at the recipient site were scored by patients on a 10-point scale as a composite visual analogue scale at the last follow-up visit. The results of the 10-point scale demonstrated very satisfactory surgical outcomes indicating HTFF may be recommended for patients with volar defects in the hand.

## Results

Table 1 presents the operative details and patient demographics. The mean harvest time for HTFF was 46 minutes. The mean flap area obtained was 10.84 cm ^2^ . Overall, 13 HTFF were elevated with the digital nerve branch, and one HTFF was elevated without the digital nerve branch due to the absence of an innervated digital nerve branch. Table 2 presents the postoperative outcomes. The follow-up period ranged from 6 to 13 months. Total flap survival was observed in 13 HTFF, and no postoperative explorations were performed due to arterial and venous insufficiency. One case of HTFF showed partial necrosis; however, after debridement of necrotic tissues, it was covered with a simple full-thickness skin graft. In all the cases, there were no complications at the donor site such as scar contractures, donor site depressions, keloids, wound dehiscence, hypersensitivity, numbness, or neuroma pain. In addition, there were no complications or complaints related to the recipient site and flap elasticity and durability were acceptable in all cases. At the last follow-up visit, the highest surgical outcome score was 10 points and the lowest surgical score of 7 points which was recorded in two patients (mean surgical outcome score, 8.5). Both patients complained of mild flap bulkiness.

**Table 1:** Patients demographics of hypothenar free flap (HTFF) with oblique design

	Location of defect	Sex/Age	Mechanism of injury	Number of included perforator	Flap size (cm ^2^ )	Operative techniques	Flap harvest time (min)
Patient 1	LIF pulp	M/36	Friction burn Bicycle TA	1	5.5 × 2.5	Innervated HTFF	47
Patient 2	LIF dorsum	M/56	Saw injury	1	5 × 2.5	Innervated HTFF	54
Patient 3	RIF pulp	M/43	Press injury	2	4.5 × 2.5	Innervated HTFF	33
Patient 4	LIF P1 volar	M/50	Press injury	1	5.5 × 2	Innervated HTFF	58
Patient 5	LLF pulp	M/27	Press injury	2	3 × 2.5	Innervated HTFF	39
Patient 6	LIF, LLF pulp	M/53	Press injury	2	5.5 × 3	HTFF	65
Patient 7	LLF P2 volar	M/71	Saw injury	2	5 × 3	Innervated HTFF	32
Patient 8	LRF, LSF pulp	M/65	Press injury	2	5 × 2.5	Innervated HTFF	59
Patient 9	LLF pulp	M/70	Press injury	2	3 × 2	Innervated HTFF	47
Patient 10	Right thumb volar	M/58	Paint gunshot injury	2	4 × 2.5	Innervated HTFF	48
Patient 11	RLF pulp	M/50	Traffic accident	2	3 × 2	Innervated HTFF	40
Patient 12	LLF P1-P2 volar	M/72	Farmyard injury	2	5 × 3	Innervated HTFF	41
Patient 13	LIF pulp	M/55	Press injury	1	3.5 × 2.5	Innervated HTFF	50
Patient 14	RRF P2 volar	M/44	Traffic accident	1	3 × 2	Innervated HTFF	42

Abbreviations: LIF, left index finger; LLF, left long finger; LRF, left ring finger; LSF, left small finger; M, male; RIF, right index finger; RLF, RIGHT long finger; RRF, right ring finger; TA, traffic accident.

**Table 2:** The operative results of hypothenar free flap (HTFF)

	Flap survival	Follow-up period (mo)	Surgical outcome score by patients (Perfect – 10 point)	Complications of donor site
Patient 1	Survival	7	9	None
Patient 2	Survival	12	9.5	None
Patient 3	Survival	6	8	None
Patient 4	Survival	10	7	None
Patient 5	Survival	9	9	None
Patient 6	Survival	7	8.5	None
Patient 7	Survival	6	8.5	None
Patient 8	Survival	6	9	None
Patient 9	Survival	10	8	None
Patient 10	Partial necrosis	6	10	None
Patient 11	Survival	11	8.5	None
Patient 12	Survival	6	9	None
Patient 13	Survival	13	7	None
Patient 14	Survival	7	8.5	None

### Cases

#### Case 1

**Fig. 3:**
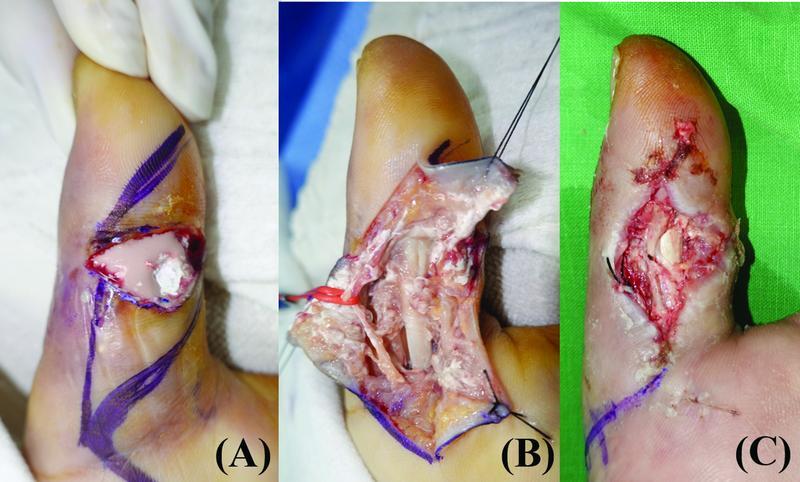
( **A** ) A 58-year-old man with infective flexor tenosynovitis and pus drainage in the right thumb. ( **B** ) Although pus drainage and extensive tenosynovectomy were performed, white-colored paint and inflammatory tissues continued to persist. ( **C** ) Postinfection control and repetitive debridement, volar soft tissue defect was seen.

**Fig. 4:**
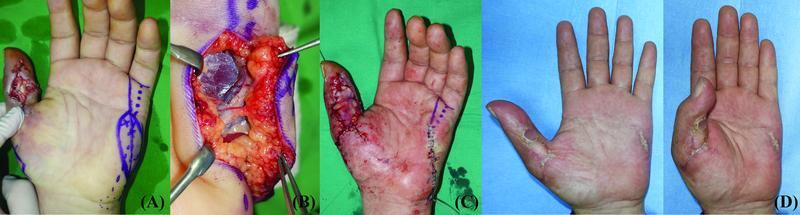
( **A** ) A 4 × 2.5 cm oblique designed hypothenar free flap was planned. ( **B** ) Two perforators from the ulnar digital artery of the little finger are included in the hypothenar free flap. ( **C** ) Postoperative results. ( **D** ) At 6 months' follow-up, no complications were observed at flap and donor site.

A 58-year-old man presented with a volar defect of the right thumb after infective tenosynovitis due to a paint gun gunshot injury ( Fig. 3 ). A 4 × 2.5 cm ipsilateral innervated HTFF with an oblique axis was planned for reconstruction of the volar side of the thumb. One artery and one vein were anastomosed. Two branches of the UDN were coapted with the radial digital nerve (RDN) in an end-to-side fashion. The donor site was closed without tension. The flap was partially necrosis since the patient was a heavy smoker; however, it was possible to completely cover the flap with a full-thickness skin graft. Mild flexor adhesion occurred at 6 months postoperatively; however, the patient was satisfied with the postoperative results both functionally and aesthetically ( Fig. 4 ).

#### Case 2

**Fig. 5:**
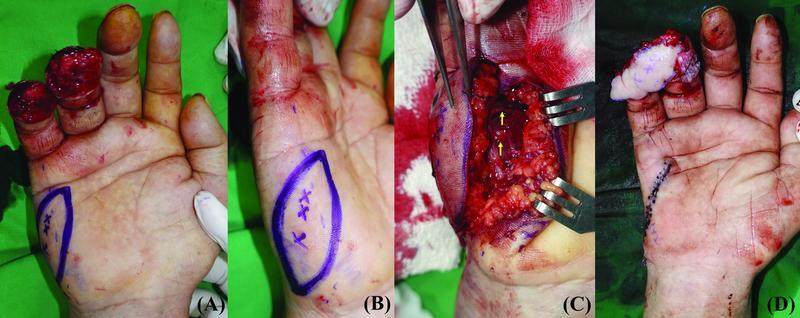
( **A** , **B** ) A 65-year-old man for whom a 5 × 3 cm oblique axis hypothenar free flap was planned for the reconstruction of pulp defects in the ring and little fingers. ( **C** ) Two perforators were observed branching from the ulnar digital artery of the little finger (arrow). ( **D** ) Postoperative results.

A 65-year-old man presented with pulp defects in the ring and little fingers after a compression injury at his workplace. A 5 × 3 cm ipsilateral innervated HTFF with an oblique axis was planned for simultaneous reconstruction of the entire pulp tissue. Two little finger UDA perforators were anastomosed with the RDA of the ring finger, and the HT branch of the basilic vein was anastomosed with the superficial vein of the thumb. One branch of the UDN was coapted with the RDN in an end-to-side fashion. The donor site was closed without tension. All of the flaps were viable with no complications such as depression scars at the donor site. At 6 months postoperatively, no complications were observed at the donor or flap sites. The patient was satisfied with the aesthetic appearance of the donor site ( Fig. 5 ).

#### Case 3

**Fig. 6:**
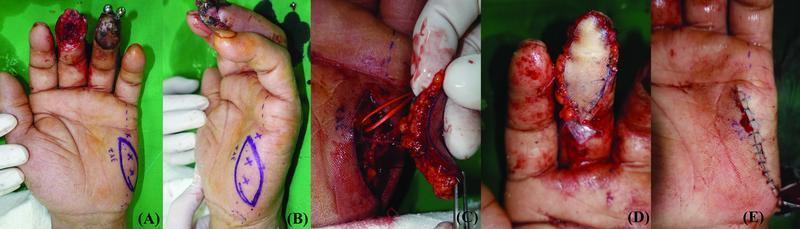
( **A** , **B** ) A 56-year-old man for whom a 3 × 2 cm oblique hypothenar free flap was planned for the reconstruction of a long finger pulp defect. ( **C** ) One perforator from the ulnar digital artery was included in the flap. ( **D** ) Postoperative results. ( **E** ) The donor site was closed without tension and depression.

A 55-year-old man presented with a pulp necrosis of the left index finger following a replantation. A 3.5 × 2.5 cm ipsilateral innervated HTFF with an oblique axis was planned. One branch of the UDN was coapted with the UDN in an end-to-side fashion. The donor site was closed without tension. All of the flaps were viable without any complications. At 6 months postoperatively, there were no complications at the donor site such as depression, keloids, pain, or hypersensitivity. In addition, the flap contour, durability, and elasticity were acceptable with a full range of motion achieved ( Fig. 6 ).

## Discussion

The essentials for a new flap design contain that is easy to reproduce, has pedicles at a consistent location, and does not cause donor site morbidity. The advantages of the HTFF design that we introduced in this study, include its simplicity and a consistently located pedicle. The perforator in the distal ulnar HT area was used for the pedicle of the flap and was located in the distal third of the flap. Studies by Omokawa et al, Uchida et al, and Han et al, have shown that the most appropriate perforator for the flap is located in the distal ulnar HT, and almost all perforators that originated from the UDA were located in the distal ulnar HT area. 3 6 7 In our study, the perforators that were located in the distal ulnar HT and branched from the UDA of the little finger were used in all of the patients.

**Fig. 7:**
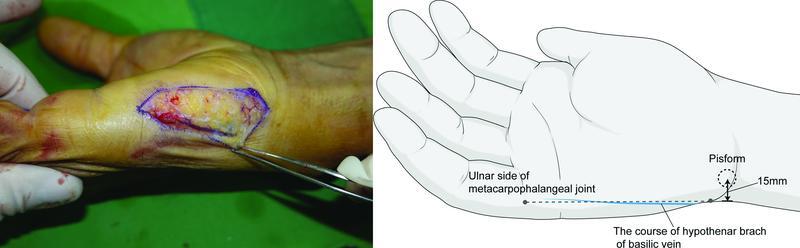
Hypothenar branch of the basilic vein coursing along the line between the ulnar side of the little finger and 15 mm on the ulnar side of the pisiform.

**Fig. 8:**
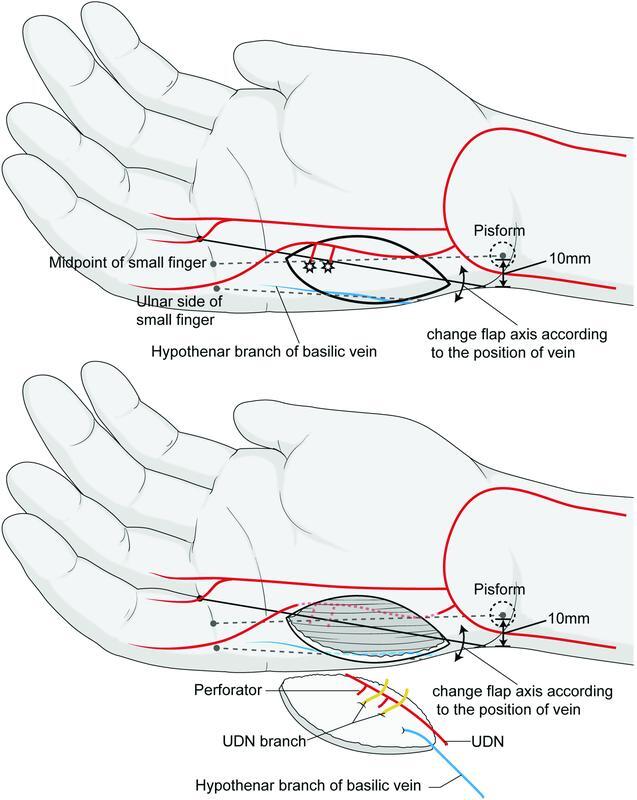
The schematic preoperative design for innervated hypothenar free flaps. Considering the position of a hypothenar branch of the basilic vein, the flap axis can be slightly adjusted to ensure abundant circulation and a long pedicle with large flap elevation.

In our study, the HT branch of the basilic vein was always included in our flap design. This vein had a large diameter and constant location, and therefore, we propose the pathway of the superficial vein. More studies, such as cadaver studies, are needed in the future that would identify suitable locations on the superficial veins. The HT branch of the basilic veins in the patients in this study were all located along the line joining the ulnar side of the MCP joint of the little finger with the ulnar side 15 mm from the center of the pisiform ( Fig. 7 ). Further, if there are any concerns about the location of the vein before harvesting the flap, the HT branch of the basilic vein can be found as it will turn bluish under the pneumatic tourniquet. If it is different from the position of the expected venous pathway, considering the position of the vein, the flap axis can be slightly changed to include this vein in the flap ( Fig. 8 ).

Recently, many methods of treating fingertip and pulp amputations with a simple method have been introduced. However, for better functional and aesthetic results, reconstruction using flaps is required. In order to use flap coverage as the first choice in fingertip or pulp reconstruction, flaps should be easily harvested and anastomosis should be performed. Many studies are trying to suggest flaps with these features. When reconstructing palmar and pulp defects of the hand and finger, various flap options, such as second toe pulp transfer, radial artery superficial palmar branch (RASP) flap, free tissue transfer flaps, homodigital reverse island flaps, thenar flaps, and cross-finger flaps are available as local flaps. 12 13 14 However, there are some disadvantages for each of these flaps, including requirement of additional anesthesia before flap harvest for the second toe pulp transfer, limitation of radial and palmar abduction, 14 wrist pillar pain for RASP flap, and the need for a secondary operation in the case of thenar and cross-finger flaps. The homodigital reverse island flap can result in scar contractures at the donor site and may require another donor site for skin grafts. 15 On the other hand, the HTFF can be harvested with glabrous tissue in one stage, under single administration of anesthesia and operative field. However, there are several drawbacks of HTFF yet; if these drawbacks are overcome, HTFF can be the ideal flap of choice for palmar tissue defect reconstructions of the hand.

The first drawback is the limitation in flap size. In most cases of hand trauma, such as workplace crush injuries, reconstruction of the entire pulp or multiple-digit pulp defects are frequently needed. Therefore, a larger flap may be required for expanding the indication of HTFF to allow sufficient coverage of palmar defects.

Second, the HTFF has a thin perforator diameter and a short pedicle. Microsurgical techniques have recently been developed to deal with vessels that are ≤ 1 mm, which have helped widen the indications for microsurgery. However, a thin vessel diameter may be challenging for a beginner flap surgeon. Furthermore, the learning curve is high, and vessel spasm can easily occur.

Finally, donor site morbidity can occur. Although the skin of the HT area has redundancy, it can cause scar contracture by generating a donor site scar perpendicular to the relaxed skin tension line (RSTL) and HT crease and donor site depression after harvesting the large flap vertically in the HT area. 9 10

To overcome these limitations, HTFF should be used to harvest larger flaps with a large pedicle diameter under single anesthesia and operative field. A number of anatomical and clinical studies have been conducted on HTFF. In 2005, Hwang et al 5 introduced a flap using a musculocutaneous perforator in the proximal HT area, while Uchida et al, 6 Han et al, 7 and Pak et al 10 conducted studies to determine the location of the perforator and nerve branch in the HT area. A further HTFF clinical study by Kim et al has provided detailed anatomical information. 8 However, these studies focused on the source pedicle vessel and anatomical position of the perforator but did not focus on how to effectively design flaps for harvesting large flaps without donor site morbidity. Therefore, in our study, some tips with a new concept of HTFF design were suggested. It was the oblique axis of the flap technique along the RSTL and providing a flap source artery and large diameter vein within the flap.

**Fig. 9:**
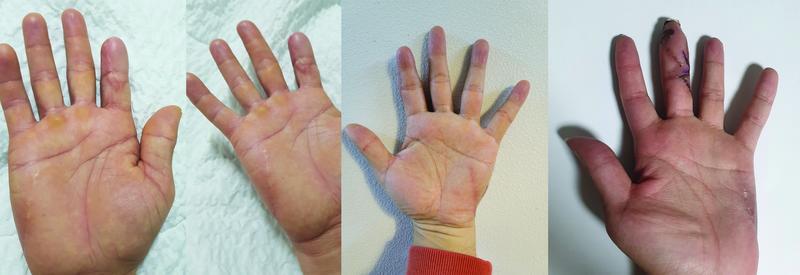
Long-term (about 8 months) postoperative donor site scar after modified oblique axis hypothenar free flap. There are not any depressed scars and contractures on the donor site. All of the patients were satisfied with the donor site aesthetically and functionally.

First, the advantages of using the oblique axis for HTFF are the flap position and the ability to close the donor site without tension. By positioning the perforator on the distal third of the HTFF, most of the flap area is located above the proximal HT muscle belly. The proximal HT area is more mobile than the distal HT area and has glabrous tissue that is similar to the distal HT area. This allows reduced tension at closure, a depressed scar with palmar sliding of dorsal skin along the RSTL, and postoperative scar contracture due to being parallel to the RSTL of the HT area and the elasticity of dorsal hand tissue. In our study, donor site closure was possible up to a size of 3 cm without tension in our study. A more mobile donor site allows the harvesting of larger flaps. This method also results in satisfactory aesthetic outcomes, with all patients found to be satisfied with the operative results with minimal donor site scars ( Fig. 9 ). In addition, by using an ulnar oblique axis flap additional distal superficial veins can be included in the flap if required.

Second, the use of a large diameter vessel for the pedicle (UDA of the small finger and HT branch of the basilic vein) has a number of advantages. The vena comitans of the digital artery can be used for venous drainage; however, this vein is not always present and can be very thin. 16 Including the perforator and UDA of the little finger enables a longer and thicker pedicle, thereby preserving the abundant circulation in the flap and decreasing the technical difficulty of anastomosis.

Recently, to expand indications and overcome the limitations of HTFF, Kodaira and Fukumoto 11 and Yamamoto et al 9 have independently posited the use of novel methods. Kodaira and Fukumoto used a distal perforator to harvest flaps including the UDA of the little finger and dorsal veins to facilitate anastomosis and obtain longer pedicles, whereas Yamamoto et al used an additional transposition flap from the ulnar side to harvest larger flaps. 9 11

Kodaira and Fukumoto's methods have the advantages of ensuring longer and thicker pedicles, but are limited in the distal HT area. They can also result in scars that are perpendicular to the distal HT crease leading to scar contractures. In addition, superficial veins in the distal HT area are thinner than those in the proximal area. Yamamoto et al's methods are advantageous for harvesting larger flaps, but require additional transposition flaps to achieve donor site closure. The donor site scar of the transposition flap will be formed longitudinally on the margin of the HT area, which can potentially become a painful scar. 9 In addition, because superficial vein on the palm side usually thinner than the dorsal side, including the dorsal side vein (HT branch of basilic vein), was better than Yamamoto et al's flap design that was mainly positioned on the palmar side.

We agree with Kodaira and Fukumoto's study, wherein they included a perforator with UDA and superficial vein, and Yamamoto et al's study, wherein the necessity of a larger flap was suggested. In our study, we incorporated both these while eliminating the disadvantages. At the last follow-up of the patients in our study, the surgical outcome score by patients was fine. It showed that the long-term operative result was fine either functionally and aesthetically. With our design, we can easily harvest a larger flap, with a thick and long pedicle with minimal donor site morbidity. HTFF can be considered as the ideal flap choice to cover almost all palmar digit defects. Fig. 8 shows our HTFF design considering the perforator, vein position, and donor site morbidity. This study is meaningful in that some tips with the new concept may aid in HTFF design by demonstrating the use of landmarks on the palmar side of the hand.

Further study is needed to gain further insights into HTFF. Innervated HTFF, which included the branch of the UDN, was used in almost all the patients in this study. The branch of UDN included in the HTFF is thin; thus, long-term follow-up is necessary to see how much nerve reinnervation is required to recover sensory deficits. In addition, since little fingers have both digital arteries, a sacrifice of the UDA will not affect circulation in the little fingers. Nevertheless, long-term follow-up is needed to determine any other complications, such as cold intolerance. Further studies should be conducted on the innervated HTFF and the use of the main vessel of the UDA of the little finger. It could allow for wider use of HTFF and result in more favorable results when used for palmar defect reconstructions.

In conclusion, our suggested tips with a new concept for HTFF elevation may have utility in the reconstruction of palmar defects, thereby increasing the application of HTFF and improving operative results. Future studies on innervated HTFF with long-term follow-up will provide further insight and determine the efficacy and long-term outcomes of HTFF in reconstructing palmar defects.
